# Assessing Cognitive Abilities Using the WAIS-IV: An Item Response Theory Approach

**DOI:** 10.3390/ijerph18136835

**Published:** 2021-06-25

**Authors:** Gomaa Said Mohamed Abdelhamid, Marwa Gomaa Abdelghani Bassiouni, Juana Gómez-Benito

**Affiliations:** 1 Quantitative Psychology Unit, Faculty of Psychology, University of Barcelona, 08035 Barcelona, Spain; juanagomez@ub.edu; 2 Group on Measurement Invariance and Analysis of Change (GEIMAC), Institute of Neurosciences, University of Barcelona, 08035 Barcelona, Spain; 3 Department of Educational Psychology, Faculty of Education, Fayoum University, Fayoum 63514, Egypt; 4 Department of Methods of Social Work, Faculty of Social Work, Fayoum University, Fayoum 63514, Egypt; mga02@fayoum.edu.eg

**Keywords:** intelligence, cognitive, WAIS-IV, item response theory, psychometric, adults, validation, adaptation, assessment

## Abstract

*Background*: The Wechsler Adult Intelligence Scale-Fourth Edition (WAIS-IV) has been adapted to 28 different cultures and there has been considerable interest in examining its structure through exploratory and confirmatory factor analysis. This study investigates item and scale properties of the Egyptian WAIS-IV using item response theory (IRT) models. *Methods*: The sample consisted of 250 adults from Egypt. The item-level and subtest statistical properties of the Egyptian WAIS-IV were established using a combination of four dichotomous IRT models and four polytomous IRT models. In addition, factor analysis was performed to investigate the dimensionality of each subtest. *Results*: Factor analysis indicated the unidimensionality of each subtest. Among IRT models, the two-parameter logistic model provided a good fit for dichotomous subtests, while the graded response model fitted the polytomous data. Most items of the Egyptian WAIS-IV showed high discrimination, and the scale was adequately informative across the levels of latent traits (i.e., cognitive variables). However, each subtest included at least some items with limited ability to distinguish between individuals with differing levels of the cognitive variable being measured. Furthermore, most subtests have items that do not follow the difficulty rank they are ascribed in the WAIS-IV manual. *Conclusions*: Overall, the results suggest that the Egyptian WAIS-IV offers a highly valid assessment of intellectual abilities, despite the need for some improvements.

## 1. Introduction

The Wechsler Adult Intelligence Scale (WAIS) is one of the most important measures developed to assess the cognitive abilities, and it has become an essential tool for a wide range of practitioners [1,2]. The latest version of the WAIS, the fourth edition [3], introduced several improvements over previous editions [4]: (1) Reduced administration time; (2) updated structure to ensure compatibility with Carroll, Cattell, and Horn’s theoretical conceptualization of intelligence; (3) addition of 53% new items; (4) improved clinical utility; and (5) revision of the subtests administered, the order of the items, a number of sample items, start-point items, stopping rule, the administration time, and bonus-point allotment.

It comprises 10 core subtests that yield four factor index scores (i.e., Verbal Comprehension, Perceptual Reasoning, Working Memory, and Processing Speed) as well as the full-scale IQ. There are also five supplemental subtests that can be used as an alternative to the core subtests in order to overcome problems such as a sudden illness or any other reason which might otherwise prevent fulfillment of the administration terms; for instance, some individuals might have problems in motor skills that leave them unable to complete the Block Design subtest, which would thus be replaced by a supplemental subtest that does not require motor skills.

The WAIS-IV manual [3] explains how to use start points, reversal rules, basal rules, and discontinue rules to calculate the total raw scores and convert them into scaled scores, under the assumption that the scale items are ordered according to their difficulty. The administration of WAIS-IV subtests relies on the discontinue rule, which aims to minimize testing time and differs for each subtest, with the examiner usually stopping the administration when the examinee obtains a score of 0 on a specified number of consecutive items [5]. The discontinue rule assumes that having reached this point, individuals will fail to complete the remaining scale items due to the increased difficulty of items relative to their ability. The discontinue rule must be applied with care during administration and it is dependent on the assumption that subtest items proceed in order of difficulty. If the subtest items are not ordered correctly based on difficulty values, the examiner may fail to administer items below the examinee’s real ability, and hence the discontinue rule will lead to misleading results.

The WAIS-IV was standardized for 2200 individuals between the ages of 16 to 90 in the United States [3]. The issue of structural validity and adaptation of the WAIS-IV has received considerable critical attention by researchers in the USA and other countries. Several recent studies explored the structure of four- and five-factor WAIS-IV models [6,7,8,9], concluding that both these models offered a good fit. However, some WAIS-IV subtests were observed to load on more than one factor. For example, Weiss et al. [9] found that Arithmetic was cross-loaded on the Working Memory, Perceptual Reasoning, and Verbal Comprehension factors based on the WAIS-IV’s normative sample of 1800 normative adults and 411 clinical adults. Abdelhamid et al. [10], who adapted the WAIS-IV for an Arabic-speaking population, preferred the four-factor model with multi-loading of the Arithmetic subtest on two factors (Verbal Comprehension and Working Memory), although the five-factor model also fitted the data well. By contrast, other authors such as Reynolds et al. [11] have reported good fit for either the four- or five-factor model with single loading of each subtest in 104 adults with intellectual disabilities from the United States. On the other hand, Bowden et al. [1] examined the factorial invariance of the WAIS-IV across different cultures in the United States and the Canadian standardization data and found that the four-factor model using the core and supplemental subtests was invariant across the samples of both countries. Gignac and Watkins [12] examined the WAIS-IV from the perspective of the bifactor model using correlation matrices of the WAIS-IV normative sample and found that this model provided a better fit than the conventional higher-order and oblique factor models (both four- and five-factor). However, some subtests were associated with weak and/or non-significant loadings on their index factor for some age groups; for instance, the Arithmetic subtest showed non-significant loadings (0.08) on the Working Memory factor for ages 20–34.

A common feature of previous research examining the factor structure of the WAIS-IV is the consideration of subtests as variables and higher constructs (e.g., Verbal Comprehension) as factors. In the present study, by contrast, we consider each subtest as a factor and its items as the variables in the factor analysis. Assessing the psychometric properties of items within each subtest is important to ensure that their location (difficulty) and discriminatory power are accurate [13]. However, despite the importance of item difficulty, the WAIS-IV Technical and Interpretive Manual does not provide values for this parameter, and previous studies of the WAIS-IV have not addressed item difficulty. Furthermore, classical test theory has been used to assess the psychometric characteristics of WAIS-IV subtests, with item difficulty being calculated using the *p*-value [3]. However, research has highlighted a number of problems with the use of classical test theory. One such problem is that the estimation of item parameters is affected by the sample used, while the estimation of person parameters is affected by the items used [14]. Given these and other limitations it has been argued that item response theory (IRT) models should be used to achieve measurement precision [15].

IRT is an important consideration in the development of psychological and educational measures [16], and it has become a powerful statistical tool for scale development and construction that provides psychometric information about every item in a scale [17,18,19,20]. It has many features that make it a preferred alternative to classical theory, and much previous research has explored its advantages over the latter [21], particularly as regards its statistical independence in estimating the individual parameter (ability θ) and the item parameters. In other words, unlike classical theory, the item parameters estimated using IRT models are invariant across different samples.

With respect to the WAIS-IV, there are two important issues which may be considered in light of the above. One is the extent to which the WAIS-IV subtests discriminate well between high and low ability, while the other is to what extent the difficulty parameter values match those proposed by the test’s developers. Accordingly, our aim in this study was to use IRT analysis to evaluate the content of each of the WAIS-IV subtests in order to gain a detailed understanding of their measurement precision. Specifically, we assess the measurement precision of each subtest using the best fit model from among the various IRT models, and also examine the extent to which the items distinguish well between the levels of different individuals. Given that IRT models assume that test items measure a single trait [22], and that the WAIS-IV developers designed each subtest to assess a single trait, it is theoretically valid to analyze each of the subscales individually. However, this study also aims to determine the extent to which items in each subtest assess the single trait being measured; we do this by analyzing each subtest using factor analysis with polychoric and tetrachoric correlations. Overall, we hypothesized that these IRT analyses would provide evidence supporting the psychometric properties of the WAIS-IV, as well as identifying strengths and weaknesses within each subtest.

## 2. Materials and Methods

### 2.1. Participants

A volunteer sample of 250 Egyptian adults was tested between 2015 and 2016. Participants ranged in age from 18 to 24 years (M = 20.65 years, SD 1.71), and 62% were female. Once informed consent had been received, each participant was individually assessed by a psychologist or educator trained to apply the WAIS-IV in accordance with the administration manual [3]. All tests were administered and scored according to the rules described in this manual. The study was approved by the Research Ethics Committee of the University of Fayoum and complied with its guidelines.

### 2.2. Instrument

The WAIS-IV used in the present study was the version for Arabic speakers, adapted and validated by Abdelhamid et al. [10,23]. It consists of 10 core subtests (Block Design, Matrix Reasoning, Visual Puzzles, Digit Span, Arithmetic, Similarities, Vocabulary, Information, Symbol Search, and Coding) that yield scores on four factors, as well as a full IQ score. The instrument also includes five supplemental subtests (Comprehension, Picture Completion, Figure Weights, Letter-Number Sequencing, and Cancelation) that can be applied as an alternative to some core subtests, although only two supplemental subtests may be used for each individual examinee. The WAIS-IV subtests can be classified into dichotomous and polytomous subtests (see Table 1). The former are scored ‘0, 1′ on all items and comprise the following subtests: (i) Visual Puzzles (26 items), Figure Weights (27 items), Matrix Reasoning (26 items), and Picture Completion (24 items), which together assess Perceptual Reasoning; (ii) Arithmetic (22 items), which is a measure of Working Memory; (iii) Information (26 items), which assesses Verbal Comprehension; and (iv) Symbol Search (60 items) and Coding (135 items), which reflect the Perceptual Speed factor. The polytomous subtests are rated using more than two score options (i.e., 0, 1, 2, etc.) and comprise: (i) Block Design (14 items), which assesses Perceptual Reasoning; (ii) Similarities (18 items), Vocabulary (30 items), and Comprehension (18 items), which measure Verbal Comprehension; (iii) Digit Span [comprising Digit Span-Forward (eight items), Digit Span-Backward (eight items), and Digit Span-Sequencing (eight items)] and Letter-Number Sequencing (10 items), which assess Working Memory; and (iv) Cancellation (two items), which reflects the Perceptual Speed factor. Item content for the adaptation into Arabic of the WAIS-IV is identical or equivalent to that of the original U.S. version: no changes were made to the non-verbal subtests (including Visual Puzzles, Figure Weights, Picture Completion, Matrix Reasoning, Symbol Search, Coding, Block Design, and Cancellation), although some items on the verbal subtests (i.e., Similarities, Vocabulary, Arithmetic, Information, and Comprehension) were adapted for the Arabic language. For the Letter-Number Sequencing subtest, the English alphabet and numerals were converted to their Arabic equivalents, taking into account alphabetical ordering during item adaptation. Numbers used for Digit Span (Forward, Backward, and Sequencing) were likewise converted to their Arabic equivalents.

Administration and scoring of the WAIS-IV was performed individually in accordance with the guidelines set out in the administration manual [3]. Each subtest begins with a set of easy items that were developed for adults with low cognitive abilities, such as those with learning difficulties. These items are not administered to individuals with typical development, except if the person responds incorrectly to the standard start items [3]. As the sample used in the present study was comprised solely of individuals with typical development, these easy items were not considered for the analysis. The Symbol Search, Coding, and Cancellation subtests were also excluded from the analysis because their focus is more on the total score in a fixed time period than on item difficulty. The supplemental subtest Comprehension was likewise excluded. Further studies are therefore required to assess the quality of these subtests. The estimated time to apply core subtests is 60–90 min, and up to two hours may be necessary in the case of all subtests.

Due to the polytomous response format and the large number of individual items corresponding to the Perceptual Reasoning (117 items), Working Memory (56 items), and Verbal Comprehension (92 items) factors, the IRT analysis at the factor level would have required an excessively large sample size. Therefore, the current analyses were conducted at the item and subtest level.

### 2.3. IRT Models

Item response theory models provide more details about the quality of items and scales and their relationship to the trait(s) measured (cognitive constructs in the present study) [24]. More specifically, IRT offers a family of models for dichotomous and polytomous data [25] which estimate two or three parameters for each item depending on the IRT model used: a slope or discrimination parameter (a), a location parameter (difficulty; b) for dichotomous items or threshold parameter (b1, b2, b3, etc.) for polytomous items, and a pseudo-guessing parameter (c). Items with higher values on the discrimination parameter discriminate well between individuals across levels of the latent trait (θ). Item threshold parameters indicate the latent trait (θ) level required to respond correctly to the item: a higher value of the difficulty or threshold parameter implies more difficult items. In some situations the examinee may just guess. Because success in responding properly depends on the quality of the wrong alternatives used, some IRT models attempt to accommodate the pseudo-guessing parameter when estimating the ability and item difficulty parameters.

For dichotomous data, Lord [26] proposed the three-parameter logistic model (3PLM), which is based on difficulty, discrimination, and pseudo-guessing parameters. The two-parameter logistic model (2PLM) described by Birnbaum [27] does not fix the discrimination parameter (a), which thus differs across items. The one-parameter logistic model (1PLM) is the simplest model for dichotomous data and it is based on a discrimination parameter that is fixed for all items. Another IRT model is the Rasch model [28], which assumes that the discrimination parameter is fixed at a value of 1. These models were extended for polytomous data. The partial credit model (PCM-R) [29] is one of the Rasch family of models and is considered the simplest model for ordered categories [30], fixing the discrimination parameter (a) at 1 for all items. The PCM-R has been adjusted to allow calculation of the item discrimination parameter for all items fitting the one-parameter model and the partial credit model (PCM-1PL) [31]. Also for polytomous data, Muraki [32] proposed the generalized partial credit model (GPCM), while Samejima [33] described his graded response model (GRM), which was an extension of the 2PLM for ordinal data.

Given that IRT required unidimensionality, we analyze each subtest using the R packages ‘polycor’ v7–8 [34] and ‘psych’ v1.6.6 [35] to conduct factor analyses. In each subtest, we use the inter-item polychoric and tetrachoric correlations, which provide the most accurate estimates of pairwise correlations and factor loadings. Next, the unidimensionality of each subtest was examined via common criteria (e.g., proportion of the variance accounted for the first factor, standardized loadings of items ≥ 0.30, scree plot).

In accordance with Penfield’s [36] recommendation to use the IRT model with the best fit to the data, the present study aims to identify the best-fitting model for each subtest of the WAIS-IV in order to determine the measurement precision. Several different methods were used to identify the best-fitting IRT model for each subtest (dichotomous or polytomous), as described in Finch and French [37]: (1) the −2 log likelihood; (2) the root mean square error of approximation (RMSEA) index [38], where the model that best fits the data has the smallest value of a RMSEA ≤0.05; (3) the Akaike information criterion (AIC), with smaller values being considered preferable; and (4) the S-$X^{2}\;$ statistic [39] for dichotomous items and a generalization of the S-$X^{2}\;$ statistic [40] for polytomous items, which in both cases estimates the amount of similarity between the observed responses and the expected frequencies for each item, and which was calculated to identify the model fit of each item at a significance level of 1% (i.e., where *p* < 0.01 indicates item misfit) [41]. For discrimination, we used the guidelines proposed by Baker and Kim [42]: 0.35 – 0.64 = low; 0.65 – 1.34 = moderate; 1.35 – 1.69 = high; ≥ 1.70 = very high, which indicate that the minimum value for retaining an item based on discrimination parameter is 0.65. In addition, we estimated item functions and test information functions (TIFs), which show at which levels of the latent trait (θ) the item and test estimates are most precise (Hambleton & Jones, 1993). Test information curve, which indicates the total of the item information curves, was plotted for each subtest to visualize the amount of information at any given level of ability θ. All the IRT analyses were performed using IRTPRO 4 [43].

## 3. Results

### 3.1. Dimensionality

Factor analysis revealed that for each subtest the first factor accounted for the largest proportion of the variance, ranging from 37% for Vocabulary to 65% for Arithmetic (see Table 2, column 2). In addition, the standardized loadings of most items were very high for the one-factor solution; However, a few items of some WAIS-IV subtests did show poor loading (i.e., loading < 0.30; see Table 2, column 3). Nonetheless, these results meet the recommendation of Reeve et al. [44] regarding the total variance that should be estimated by the first factor and suggest that each of the WAIS-IV subtests is indeed unidimensional. For more information, the complete loading parameters are listed in Tables S1 and S2 (FA column) in the Supplementary Materials.

### 3.2. IRT Results

Table 3 and Table 4 compare the fit statistics of IRT models for the WAIS-IV subtests, using the likelihood statistic (−2log.Lik), AIC, and RMSEA.

#### 3.2.1. For Dichotomous Data

Examination of the RMSEA statistic showed that the 2PLM and 3PLM produced an adequate fit in comparison with the RM and 1PLM (see Table 3). Note also that the lowest set of AIC values was found for the 2PLM.

The S-$X^{2}\;$ values likewise revealed that the 2PLM fitted well with the WAIS-IV items, as compared with the 3PLM model, although the former did not fit five items from Picture Completion and two items from Visual Puzzles, Matrix Reasoning and Figure Weights (see Table 5 and Table S1). According to the model- and item-level results, the 2PLM provided the best fit for the six dichotomous subtests of the WAIS-IV, supporting the use of this model. Consequently, and as recommended by Penfield [36], we report the item-level parameters based on a 2PLM for the WAIS-IV subtests. Table 5 presents the summary results for each subtest within the framework of the 2PLM, while the complete estimation parameters using the 2PLM are set out in Table S1 in the Supplementary Materials.

Most items of the six dichotomous subtests yielded high discrimination values (according to the guidelines of Baker and Kim [42]), confirming that these items differentiate well between individuals with different levels of the trait being measured. However, some items did not meet the minimum discrimination value (i.e., a < 0.65), for example, items 6 and 8 in Matrix Reasoning. Values of the difficulty parameter indicated that all dichotomous subtests can estimate well a wide range of individual ability levels (θ), from low (learning disability) to high (gifted). For example, the difficulty parameter for Picture Completion ranged from −3.52 to 3.32 logit, indicating the spread of items across the levels of the trait being measured. The items of each dichotomous subtest were ordered based on the difficulty parameter (b), according to which, items with a higher (b) value are more difficult (Table S1). Interestingly, each of the dichotomous subtests includes some items that do not match the original ranking suggested in the WAIS-IV manual; consider, for instance, the Arithmetic subtest, where item 19 was easier than items 16, 17, and 18.

#### 3.2.2. For Polytomous Data

As shown in Table 4, the GRM and GPCM showed better fit than did the PCM-1PL and PCM-R for all subtests. Based on the RMSEA and AIC results, the GRM and GPCM provide a better fit than do the PCM-R and PCM-1PL for the WAIS-IV subtests; the GRM and GPCM had the lowest set of RMSEA and AIC values for the polytomous subtests.

Results for the S-$X^{2\;}$ statistic likewise indicated that both the GPCM and GRM fitted the data well, although these models did show misfit for some items from the subtests Similarities and Vocabulary (see Table 5 and Table S2). If we consider the Similarities subtest, for example, two items and four items, respectively, had a poor fit under the GPCM and GRM models. Overall, the model- and item-level fit results indicate that the GRM is the preferred option for polytomous WAIS-IV subtests. Consequently, Table 5 only summarizes the item parameters estimated for polytomous WAIS-IV subtests within the framework of the GRM, although the complete estimation parameters are displayed in Table S2 in the Supplementary Materials.

It should be noted that most polytomous items have high discrimination values, indicating that these items have a strong relationship with the latent trait being measured and that they can distinguish well between different levels of individuals on the target variable. For instance, the discrimination value for item 9 of the Letter-Number Sequencing subtest was around 3, indicating excellent discrimination according to the guidelines of Baker and Kim [42]. However, low discrimination was also found for some items (e.g., item 9 from Vocabulary), and these should therefore be reviewed.

The threshold values of most items covered different levels of the latent trait being measured (Table S2). For example, item mean threshold values for Similarities ranged from ‒3.26 (item 6) to 2.56 (item 18). However, it should be noted that the range of mean threshold values for some subtests, such as Block Design, is somewhat limited (1.40:2.19 logit), although this may be due to the exclusion of the easiest items from our analysis. The items of each polytomous subtest were ordered using their mean thresholds (Table S2), according to which, items with a higher mean threshold are more difficult. The results here showed that some items from the subtests Block Design, Similarities, and Vocabulary were ranked differently to the rank they are assigned in the WAIS-IV manual. For example, on Similarities, item 10 was easier than item 9, and item 14 was easier than items 11, 12, and 13. By contrast, the rankings obtained for items on the Digit Span and Letter-Number Sequencing subtests are similar to those in the WAIS-IV manual.

#### 3.2.3. Measurement Precision 

**Figure 1:**
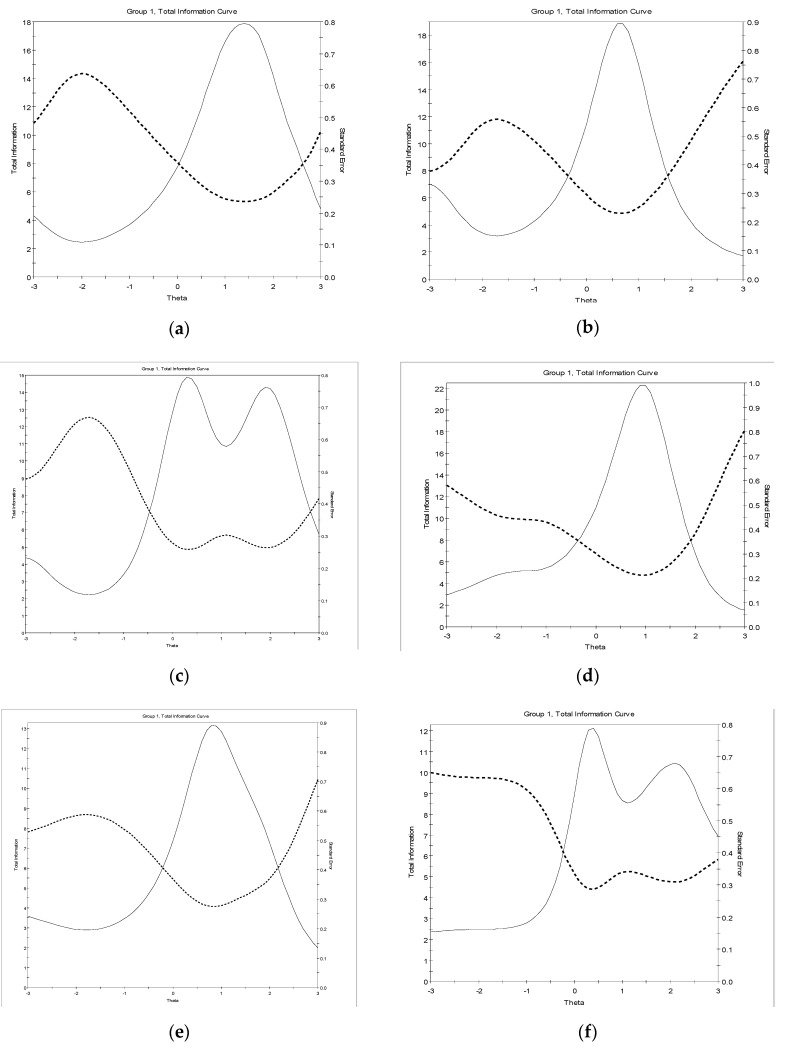

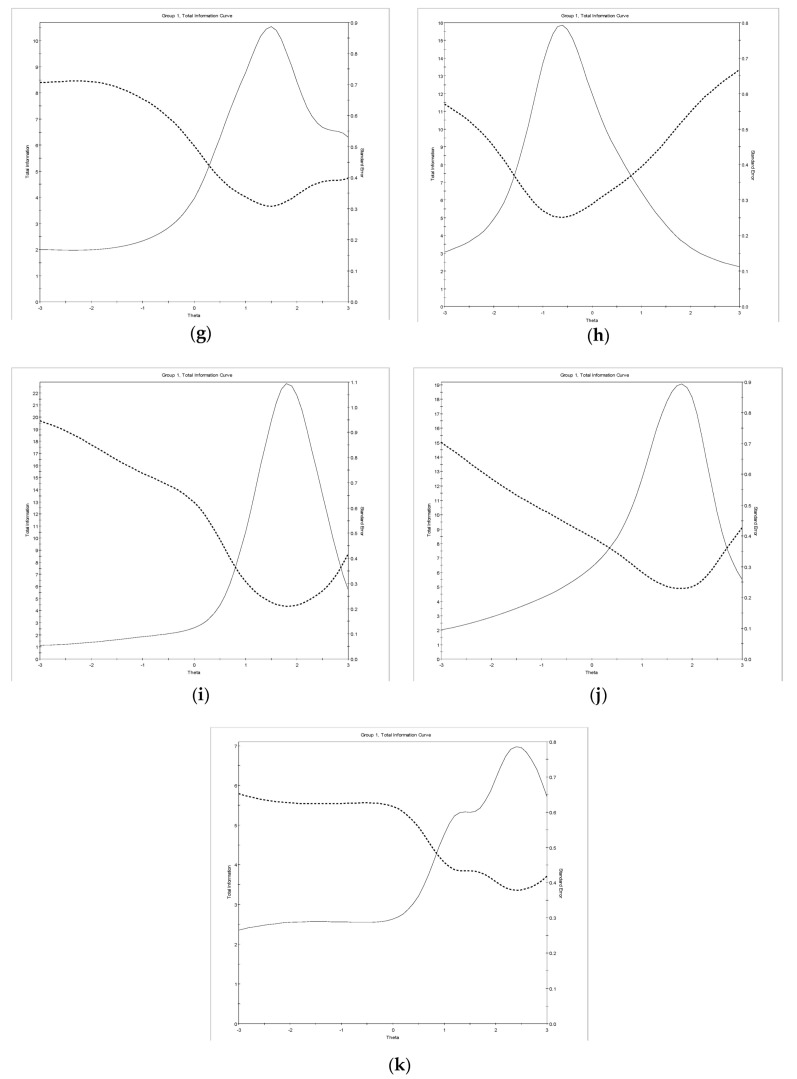
Test information function curve for dichotomous WAIS-IV subtests: (**a**) Visual Puzzles; (**b**) Matrix Reasoning; (**c**) Picture Completion; (**d**) Figure Weights; (**e**) Arithmetic; (**f**) Information. Test information function curve for polytomous WAIS-IV subtests: (**g**) Similarities; (**h**) Vocabulary; (**i**) Block Design; (**j**) Digit Span; (**k**) Letter-Number Sequencing.

Figure 1a–j plot TIFs, which show the amount of precision and reliability for each level of the latent trait (θ)  being assessed by the WAIS-IV subtests. For each of the WAIS-IV subtests, except Vocabulary, the TIFs curves were negatively skewed and most information was gathered above the mean latent trait levels (at θ = 0.3 or above). Vocabulary is the only subtest that was most reliable and precise below the mean θ, at around θ = −0.6. A possible explanation for this is that larger discrimination (a) values (mostly > 2) are associated with positive difficulty (b) values whereas this is not the case with Vocabulary. Nevertheless, the amount of information was at least 3 or above across ability levels between θ = −1 and θ = 2 for most of the WAIS-IV subtests, the exceptions being Similarities, Block Design, and Letter-Number Sequencing within the latent levels between θ = 0 and θ = 3. Overall, these TIF results confirm that the WAIS-IV is adequately informative for different levels of the trait being measured (i.e., cognitive variables).

## 4. Discussion

The present study has established the item-level and subtest statistical properties of the WAIS-IV, as adapted for an Arabic-speaking population, using a combination of four dichotomous IRT models (i.e., RM, 1PLM, 2PLM, and 3PLM) and four polytomous IRT models (i.e., PCM-R, PCM-1PL, GPCM, and GRM). Overall, the item analysis indicated that the WAIS-IV is an adequate tool for assessing adult intelligence in another culture (in this case, the Egyptian), with only minor modifications on some items. These results are consistent with other studies such as that by Lazarevic, Knezevic, Mitic, and Djuric-Jocic [45], who adapted the WAIS-IV for the Serbian population.

Another important finding was that the GPCM and GRM showed a better fit than the PCM-R and PCM-1PL for the polytomous WAIS-IV subtests. This finding is consistent with that of Baker et al. [46], who reported a better fit of the GRM in comparison with the PCM-R. In accordance with these findings, we estimated item parameters using the 2PLM for dichotomous data and the GRM for polytomous data. Based on the guidelines of Baker and Kim [42], the results showed high discrimination values for most of the WAIS-IV items (specifically, for around 70% of the total items). The IRT results also indicate, first, that the WAIS-IV subtests are able to differentiate between adults with different levels of the cognitive variable being measured, and second, that they gather information especially for adults whose intellectual ability is greater than average. Six subtests were observed to gather maximum information with peaks above +1 θ, with four subtests displaying peaks below +1 θ and above average θ, and only one subtest having a peak below average θ. These subtests with peaks above +1 θ are particularly appropriate for determining the level of adults with high cognitive abilities. It should be noted, however, that most of the WAIS-IV subtests had at least some items that demonstrated a limited ability to differentiate between individuals with differing levels of the latent trait being measured.

A total of 25 items did not fit the preferred IRT model (2PLM and GRM), 14 of which (56%) were from the polytomous subtests. Caution should therefore be exercised when decisions related to individual differences are made on the basis of all WAIS-IV items, including those showing low discrimination and/or model misfit. We recommend that these items be reviewed.

The original WAIS-IV manual assumes that items are ordered ascendingly according to their difficulty, and it relies on the discontinue rule during administration. The rationale is that because items are administered in what is assumed to be an ascending order of difficulty, the administration time can be reduced by applying the discontinue rule. The present analysis has suggested an ordering for the items of each WAIS-IV subtest based on the difficulty parameter estimated under IRT models. Somewhat surprisingly, this analysis revealed a number of differences in subtest item rank between the Arabic adaptation of the WAIS-IV and the original US version, even though the original subtest structures have been preserved as far as possible. In fact, the original item rank was preserved in only two subtests of the Arabic adaptation (i.e., Digit Span and Letter-Number Sequencing). Consequently, we would argue that the item ordering should be revised in order to address some of the possible cultural factors underlying this result. If we consider the Information subtest, for instance, most of the items relate to the Western canons of geography, science, history, and literature. Item 5, for example, refers to Martin Luther King, who is less well known among the Egyptian population, and this is reflected in the fact that this item is ranked 20st in terms of difficulty in the present analysis. There is, therefore, a definite need for substituting “Martin Luther King” with a more context-relevant equivalent to improve the scale. By contrast, item 10 refers to Cleopatra, an important figure in Egyptian history, and this item was here ranked 6th in terms of difficulty. Regarding nonverbal subtests, let us consider Visual Puzzles as an example. Here the difficulty of item 14 was ranked 17th, with a logit value of 1.08, whereas the difficulty of items 15, 16, and 17 yielded logit values of 0.65, 0.79, and 0.82, respectively, which means that item 14, in our sample, was more difficult than any of these items.

From an empirical perspective, this means that individuals may be asked to discontinue before they have the opportunity to respond to items that are in fact within their ability, and on which they would be expected to answer correctly. Consequently, our results, while preliminary, suggest that WAIS-IV items need to be re-sequenced for the Arabic-speaking population in order to yield more accurate scores. This is consistent with the conclusion reached by Abdelhamid et al. [23] and Suwartono et al. [47], who likewise suggested that the item order proposed in the U.S. WAIS-IV manual was inappropriate for, respectively, the Egyptian and the Indonesian population. However, it is also necessary to take into account the different characteristics of the samples used. As such, these results suggest that further developments of the WAIS should consider using IRT models in order to achieve more accurate estimates of item difficulty, and hence a more appropriate item ordering.

The unidimensionality assumption was confirmed by our results, indicating that each WAIS-IV subtest measures one latent trait, although there were a few items that showed poor loading on the first factor. While these findings provide additional support for the one-factor structure of each subtest, as previously reported by Reynolds et al. [11] and Gignac and Watkins [12], we nevertheless recommend that the items with poor loading on the target factor be reviewed.

Since our findings are limited to an Egyptian sample they cannot be generalized to other cultures, neither that of the USA, where the WAIS was developed, nor even that of other Arab countries. A task for future research would therefore be to assess the adequacy of the WAIS-IV in various cultural samples using IRT models. Another limitation of our study is that due to the difficulty of application, the sample comprised a narrow age range of adults. Thus, the present results should only be limited to the age range of 18–24. Future studies should seek to recruit adults of all ages in Egypt. Finally, although a few studies have examined the structure of the WAIS-IV in clinical samples using exploratory and confirmatory factor analysis [48,49], we would argue that the clinical utility of the WAIS-IV requires further investigation in terms of item-level analysis using IRT models.

## 5. Conclusions

In conclusion, the current study provides new evidence regarding the utility and measurement precision of the WAIS-IV, as adapted for Arabic speakers and particularly for the Egyptian community. Importantly, the results of our item analysis suggest a number of areas where improvements could be made. First, the items of some subtests could be reordered according to the difficulty parameter. Second, some items which showed low loading or misfit to IRT models, such as item 8 on the Similarities subtest, should be revised. Misfitting items might also be excluded when developing shortened versions of the WAIS-IV, such as those discussed by Meyers et al. [50]. Finally, we would argue that the aforementioned problematic issues could be avoided by using IRT models when developing the next version of the WAIS.

## Figures and Tables

**Table 1:** Summary of the WAIS-IV subtests.

Factor	Subtest	Number of Items	Type of Items	Scoring (Points)
Perceptual Reasoning	Block Design	14	polytomous	Items 1–4 score (0, 1, 2) Items 5–8 score (0, 4) Items 9–14 score (0, 4, 5, 6, or 7)
	Visual Puzzles	26	dichotomous	0, 1
	Matrix Reasoning	26	dichotomous	0, 1
	Picture Completion *	24	dichotomous	0, 1
	Figure Weights *	27	dichotomous	0, 1
Verbal Comprehension	Information	26	dichotomous	0, 1
	Similarities	18	polytomous	0, 1, 2
	Vocabulary	30	dichotomous polytomous	Picture items 1–3 score (0, 1) Verbal items 4–30 score (0, 1, 2)
	Comprehension *	18	polytomous	0, 1, 2
Working Memory	Arithmetic	22	dichotomous	0, 1
	Digit Span (Forward, Backward, and Sequencing)	24	polytomous	The item score is calculated as the total score of the two trials (each trial is scored with 0 or 1 points).
	Letter-Number Sequencing	10	polytomous	The item score is defined as the total score of the three trials (each trial is corrected with 0 or 1 points).
Perceptual Speed	Symbol Search	60	dichotomous	0, 1
	Coding	135	dichotomous	0, 1
	Cancellation *	2	polytomous	Maximum total raw score 72

Note. An asterisk * means supplemental subtests.

**Table 2:** Variance explained by the first factor and items with poor loading for each subtest of the WAIS-IV.

Subtest	Variance (%) Explained by First Factor	Items with Poor Loading (<0.30)
Visual Puzzles	53%	-
Matrix Reasoning	46%	three items (6, 7, 11)
Picture Completion	42%	three items (6, 8, 9)
Figure Weights	54%	-
Information	49%	one item (11)
Arithmetic	65%	-
Block Design	62%	-
Similarities	40%	one item (7)
Vocabulary	37%	five items (6, 7, 9, 10, 11)
Digit Span	38%	one item (B3)
Letter-Number Sequencing	49%	2 items (5, 6)

**Table 3:** AIC, M2, RMSEA, and LRT analysis for dichotomous WAIS-IV subtests.

Subtest	Model	AIC	RMSEA	−2log.Lik
Visual Puzzles	RM	3200.6	0.08	3156.62
	1PLM	3127.8	0.02	3081.88
	2PLM	3114.48	0.01	3038.48
	3PLM	3285.2	0.04	3153.27
Matrix Reasoning	RM	4494.7	0.06	4448.76
	1PLM	4468.2	0.05	4420.20
	2PLM	4347.16	0.04	4263.16
	3PLM	4476.5	0.00	4338.57
Picture Completion	RM	2216.6	0.10	2170.64
	1PLM	2178.5	0.07	2130.58
	2PLM	2129.38	0.05	2049.38
	3PLM	2292.8	0.00	2154.80
Figure Weights	RM	2508.1	0.08	2464.08
	1PLM	2448.1	0.07	2402.08
	2PLM	2402.94	0.07	2322.94
	3PLM	2557.6	0.00	2425.64
Information	RM	3008.6	0.08	2962.60
	1PLM	2976.8	0.06	2928.80
	2PLM	2914.78	0.04	2834.78
	3PLM	3098.8	0.00	2960.87
Arithmetic	RM	2688.5	0.08	2654.54
	1PLM	2595.9	0.03	2559.96
	2PLM	2574.7	0.02	2510.73
	3PLM	2668.7	0.00	2568.75

Note. RMSEA = root mean square error of approximation; AIC = Akaike information criterion; log.Lik = logarithm likelihood value; RM = Rasch model; 1PLM = one-parameter logistic model; 2PLM = two-parameter logistic model; 3PLM = three-parameter logistic model.

**Table 4:** AIC, M2, RMSEA, and LRT analysis for polytomous WAIS-IV subtests.

Subtest	Model	AIC	RMSEA	−2log.Lik
Similarities	PCM-R	3575	0.06	3523.00
	PCM-1PL	3566.3	0.06	3512.30
	GPCM	3450.2	0.03	3372.23
	GRM	3515.50	0.05	3443.50
Vocabulary	PCM-R	7429.6	0.06	7329.62
	PCM-1PL	7421	0.05	7319.01
	GPCM	7155.8	0.03	7005.88
	GRM	7259.76	0.00	7121.76
Block Design	PCM-R	1791.7	0.06	1749.76
	PCM-1PL	1789.2	0.05	1745.22
	GPCM	1757.3	0.03	1703.37
	GRM	1732.4	0.00	1678.46
Digit Span	PCM-R	4568.6	0.07	4504.69
	PCM-1PL	4567.5	0.07	4501.53
	GPCM	4551.6	0.08	4455.65
	GRM	4546.6	0.07	4450.64
Letter-Number Sequencing	PCM-R	1447	0.06	1413.04
	PCM-1PL	1471.9	0.07	1437.91
	GPCM	1423.4	0.05	1375.45
	GRM	1457.00	0.1	1415.00

Note. RMSEA = root mean square error of approximation; AIC = Akaike information criterion; log.Lik = logarithm likelihood value; PCM-R = partial credit model-Rasch model; PCM-1PL = one-parameter partial credit model; GPCM = generalized partial credit model; GRM = graded response model.

**Table 5:** Item misfit to IRT models, and range of difficulty parameter values for each subtest of the WAIS-IV.

Subtest	Item Misfit $(S-X^{2}$; *p*-Value < 0.01) Using IRT Model		−2log.Lik
Dichotomous subtests	2PLM	3PLM	*b_j_* (Min:Max) using 2PLM
Visual Puzzles	10, 17	10, 17, 23, 26	−3.43:2.57
Matrix Reasoning	20, 23	20, 22, 23	−3.84:1.99
Picture Completion	8, 10, 11, 12, 16	3, 8, 9, 10, 11, 12, 13, 16,19	−3.52:3.32
Figure Weights	17, 22	16, 17, 20, 21, 25, 26	−3.54:1.57
Information	-	15, 16, 17, 23	−3.99:3.67
Arithmetic	-	-	−3.78:2
Polytomous subtests	GPCM	GRM	Mean threshold *b_ik_* (Min:Max) using GRM
Similarities	8, 13	7, 8, 15, 16	−3.26:2.56
Vocabulary	11, 13, 15, 17, 18, 22, 26, 28	10, 11, 13, 15, 17, 18, 22, 26, 27, 28	−3.16:3.01
Block Design	-	-	1.40:2.19
Digit Span	-	-	−4.35:2.58
Letter-Number Sequencing	-	-	−3.53:2.69

Note. 2PLM = two-parameter logistic model; 3PLM = three-parameter logistic model; GPCM = generalized partial credit model; GRM = graded response model.

## Data Availability

The data presented in this study are available on request from the corresponding author.

## Supplementary Materials

The following are available online at https://www.mdpi.com/article/10.3390/ijerph18136835/s1, Table S1: Item loadings, fit statistics, and item parameters for dichotomous WAIS-IV subtests, Table S2: Item loadings, fit statistics, and item parameters for polytomous WAIS-IV subtests.
