# Supplementary material for: Assessing Cognitive Abilities Using the WAIS-IV: An Item Response Theory Approach
Source: Int J Environ Res Public Health. 2021 Jun 25;18(13):6835. doi: 10.3390/ijerph18136835 (PMC8297006; doi:10.3390/ijerph18136835)
Supplement: Supplementary file 1 [file ijerph-18-06835-s001.zip › ijerph-1234778-supplementary.pdf]

Table S1. Item loadings, fit statistics, and item parameters for dichotomous WAIS-IV subtests.

I	FA	p-value S-X^2		2PLM		R*	I	FA	p-value S-X^2		2PLM		R*
		2PLM	3PLM	*a*	*b*				2PLM	3PLM	*a*	*b*	
Visual Puzzles							Matrix Reasoning						
5	.80	.78	.07	2.47	-3.43	5	4	.85	.71	.65	3.51	-3.23	7
6	.80	.80	.07	2.74	-3.31	6	5	.98	.493	.14	3.22	-2.8	8
7	.66	.236	.236	1.04	-3.18	7	6	-.13	.120	.464	.58	-3.84	4
8	.42	.249	.158	1	-1.37	8	7	.23	.354	.129	.99	-3.26	6
9	.68	.226	.110	1.27	-.41	10	8	.41	.563	.507	.57	-1.72	10
10	.70	.000*	.000*	1.66	-.63	9	9	.48	.416	.353	.97	-3.42	5
11	.76	.402	.196	1.39	-.19	11	10	.58	.130	.330	.58	-2.55	9
12	.60	.540	.297	1.91	.17	12	11	.29	.576	.443	.66	-1.17	11
13	.79	.791	.312	1.99	.2	13	12	.55	.387	.174	.85	-1.17	12
14	.43	.010	.030	1.65	1.08	17	13	.75	.512	.182	1.55	-.68	13
15	.81	.201	.180	2.36	.65	14	14	.66	.177	.053	.99	-.68	14
16	.82	.235	.118	2.47	.79	15	15	.65	.077	.016	1.24	-.11	15
17	.80	.003*	.002*	2.74	.82	16	16	.76	.039	.011	1.64	-.08	16
18	.88	.186	.081	3.13	1.1	18	17	.82	.598	.302	2.13	-.04	17
19	.85	.163	.206	3.35	1.17	19	18	.78	.517	.305	2.24	.48	19
20	.67	.057	.027	2.94	1.57	20	19	.81	.080	.039	2.82	.44	18
21	.83	.200	.443	1.65	2	25	20	.78	.000*	.000*	2.56	.53	20
22	.79	.377	.328	1.91	1.9	22	21	.78	.025	.012	2.81	.69	22
23	.62	.042	.007*	2.76	1.97	24	22	.82	.011	.008*	3.51	.59	21
24	.69	.179	.151	2.36	1.93	23	23	.77	.009*	.008*	3.22	.69	23
25	.77	.327	.215	3.87	1.73	21	24	.79	.699	.381	3.37	.89	24
26	.62	.210	.009*	3.35	2.57	26	25	.63	.159	.077	2.56	1.34	25
							26	.58	.502	.087	1.79	1.99	26
Picture Completion							Figure Weights						
I	FA	p-value S-X^2		2PLM		R*	I	FA	p-value S-X^2		2PLM		R*
		2PLM	3PLM	*a*	*b*				2PLM	3PLM	*a*	*b*	
2	.58	.370	.11	2.89	-2.97	4	6	.38	.026	.091	.8	-2.8	7
3	.65	.510	.009*	1.90	-3.52	2	7	.86	.488	.072	1.37	-3.54	6
4	.68	.165	.126	1.16	-3.38	3	8	.56	.201	.011	.8	-2.31	8
5	.81	.195	.099	.66	-.62	6	9	.91	.028	.015	2.27	-1.82	10
6	.27	.014	.010	.76	-1.15	5	10	.46	.086	.084	1.28	-2.22	9
7	.73	.080	.039	.76	-.59	7	11	.78	.259	.093	1.82	-1.61	11
8	.20	.003*	.001*	1.00	.33	12	12	.45	.184	.219	.87	-1.57	12
9	.14	.015	.005*	1.97	.01	9	13	.71	.336	.156	1.23	-1.36	13
10	.83	.005*	.001*	2.89	-.18	8	14	.54	.259	.015	1.05	-.2	15
11	.33	.004*	.005*	2.06	.78	14	15	.74	.068	.029	1.9	-.3	14
12	.85	.007*	.008*	2.69	.26	11	16	.64	.055	.004*	1.44	.26	18
13	.41	.036	.009*	3.98	.15	10	17	.69	.000*	.004*	2.79	-.17	16
14	.76	.366	.115	3.94	.49	13	18	.80	.026	.148	2.42	.25	17
15	.44	.089	.150	2.28	1.82	19	19	.88	.128	.133	3.61	.42	19
16	.40	.006*	.003*	1.98	1.58	16	20	.82	.274	.005*	3.61	.97	21
17	.74	.177	.136	2.19	1.39	15	21	.79	.019	.001*	3.71	.72	20
18	.60	.135	.101	1.97	1.64	17	22	.74	.002*	.017	2.96	1.2	24
19	.61	.019	.001*	3.42	1.98	20	23	.82	.029	.123	3.28	1.11	23
20	.99	.345	.163	2.69	1.77	18	24	.83	.278	.176	3.71	.99	22
21	.99	.086	.028	2.06	2.48	23	25	.86	.236	.009*	2.79	1.28	25
22	.66	.128	.052	2.92	2.32	22	26	.75	.022	.000*	2.65	1.57	27
23	.99	.214	.124	2.28	2.05	21	27	.81	.138	.091	2.79	1.5	26
24	.66	.12	.066	1.98	3.32	24							

I	FA	p-value S-X^2		2PLM		R*	I	FA	p-value S-X^2		2PLM		R*
		2PLM	3PLM	*a*	*b*				2PLM	3PLM	*a*	*b*	
Information							Arithmetic						
I	FA	p-value S-X^2		2PLM		R*	I	FA	p-value S-X^2		2PLM		R*
		2PLM	3PLM	a	b				2PLM	3PLM	a	b	
4	.84	.181	.31	1.26	-3.99	4	6	.60	.39	.41	1.98	-3.78	6
5	.51	.388	.866	1.1	2.36	20	7	.73	.49	.39	2.00	-3.76	7
6	.45	.134	.198	.67	-2.77	5	8	.61	.606	.201	1.89	-2.75	8
7	.84	.388	.451	1.29	-2.14	8	9	.73	.381	.162	1.03	-1.76	10
8	.59	.203	.102	.67	.69	13	10	.84	.221	.116	1.07	-2.11	9
9	.70	.693	.418	1.17	-.47	9	11	.78	.301	.116	1.27	-.39	12
10	.90	.170	.146	1.26	-2.69	6	12	.85	.628	.735	1.39	-.72	11
11	.26	.161	.071	1.08	2.96	21	13	.89	.295	.066	1.50	-.11	13
12	.67	.241	.172	.78	-2.6	7	14	.95	.216	.098	2.02	.23	14
13	.55	.365	.144	1.47	-.02	10	15	.81	.200	.062	2.40	.41	15
14	.84	.173	.059	1.85	1.19	15	16	.78	.256	.107	2.13	.81	17
15	.44	.018	.004*	2	.73	14	17	.78	.705	.415	2.85	.89	19
16	.87	.018	.001*	3.91	.31	11	18	.89	.437	.230	3.20	.84	18
17	.96	.039	.002*	3.91	.33	12	19	.95	.732	.646	3.00	.76	16
18	.31	.025	.027	2.11	3.04	22	20	.81	.122	.100	2.80	1.63	20
19	.75	.025	.016	2.14	1.35	16	21	.78	.293	.239	2.64	1.73	21
20	.86	.422	.449	2.51	1.54	17	22	.78	.141	.210	2.61	2.00	22
21	.83	.352	.232	3.06	1.83	18							
22	.30	.205	.039	1.42	3.37	24							
23	.70	.334	.007*	3.59	2.31	19							
24	.72	.151	.025	1.47	3.5	25							
25	.77	.534	.022	2	3.13	23							
26	.78	.020	.017	1.85	3.67	26							

Note. I= item, *a* = discrimination parameter; *b* = difficulty parameter; FA = item loading in factor analysis, R* = rank; 2PLM = two-parameter logistic model; 3PLM = three-parameter logistic model

*p < .01

Table S2. Item loadings, fit statistics, and item parameters for polytomous WAIS-IV subtests.

I	FA	PCM-R	PCM-1PL	GPCM	GRM	GRM						
		p-value S-X^2				*a*	*b1*	*b2*	*b3*	*b4*	Mean threshold	Rank*
Similarities												
6	.40	.274	.479	.061	.371	1.30	-3.32	-3.19			-3,26	6
7	.15	.021	.073	.573	.000*	1.15	-3.91	-.31			-2.11	7
8	.45	.000*	.000*	.006*	.006*	.65	-1.31	-.77			-1.04	8
9	.43	.117	.354	.584	.640	.75	-1.04	1.40			.18	10
10	.50	.009*	.093	.523	.446	.80	-.76	-.09			-.43	9
11	.62	.262	.315	.211	.609	1.30	.82	2.54			1.68	15
12	.64	.348	.381	.328	.372	1.30	1.12	1.35			1.24	12
13	.58	.001*	.002*	.005*	.0102	1.31	1.25	1.89			1.57	14
14	.83	.003*	.000*	.024	.028	3.18	.75	1.38			1.07	11
15	.88	.004*	.000*	.019	.001*	1.15	.88	1.60			1.24	13
16	.68	.019	.005*	.023	.005*	2.36	1.70	2.16			1.93	16
17	.84	.137	.041	.672	.428	3.18	1.55	3.01			2.28	17
18	.74	.154	.058	.183	.205	1.85	1.77	3.35			2.56	18
Vocabulary												
6	.16	.000*	.000*	.015	.026	.76	-2.98	-2.25			-2.62	10
7	.09	.001*	.000*	.025	.057	.86	-3.80	-2.24			-3.02	7
8	.33	.005*	.022	.647	.580	.65	-3.53	-2.78			-3.16	6
9	.26	.000*	.000*	.029	.062	.38	-2.69	1.54			-.58	14
10	.25	.000*	.000*	.014	.000*	.79	-3.29	-.83			-2.06	11
11	.26	.000*	.000*	.009*	.004*	.90	-2.81	-2.52			-2.67	9
12	.30	.011	.020	.235	.021	1.06	-3.02	-2.54			-2.78	8
13	.60	.000*	.014	.009*	.006*	1.04	-.59	-.47			-.53	19
14	.44	.000*	.053	.042	.042	.65	.96	1.21			1.09	27
15	.54	.000*	.004*	.000*	.005*	.97	-.96	-.16			-.56	15
16	.79	.136	.126	.116	.062	.86	-1.75	-.99			-1.37	12
17	.44	.000*	.000*	.000*	.002*	.91	1.09	1.53			1.31	29
18	.67	.009*	.000*	.005*	.009*	.79	-1.24	.16			-.54	17
19	.66	.257	.401	.301	.396	.90	-.49	-.20			-.35	21
20	.82	.148	.094	.079	.092	2.07	-.67	-.44			-.56	16
21	.96	.019	.000*	.188	.228	3.50	-.91	-.71			-.81	13
22	.77	.005*	.018	.000*	.001*	2.18	-.87	1.04			.09	24
23	.70	.294	.379	.273	.372	1.06	-.47	-.04			-.26	22
24	.67	.192	.212	.098	.186	.76	.25	.45			.35	26
25	.92	.082	.029	.065	.028	3.20	-.59	-.48			-.54	18
26	.86	.000*	.000*	.000*	.000*	2.35	-.79	-.21			-.50	20
27	.80	.002*	.003*	.011	.004*	2.37	-.50	.45			-.03	23
28	.59	.001*	.000*	.000*	.000*	1.26	-.31	2.77			1.23	28
29	.37	.022	.026	.015	.015	.77	2.97	3.04			3.01	30
30	.70	.162	.222	.104	.064	2.13	.13	.18			.16	25
Block Design												
9	.44	.006*	.0001*	.092	.157	1.05	-1.06	.72	1.94	4.01	1.40	9
10	.72	.120	.112	.103	.161	1.90	.06	1.37	1.87	3.21	1.63	11
11	.75	.029	.028	.058	.107	2.34	1.23	2.04	2.14	2.90	2.08	13
12	.91	.046	.184	.0101	.082	4.10	1.08	1.65	1.84		1.52	10
13	.91	.351	.390	.502	.418	4.98	1.43	1.80	2.03		1.75	12
14	.87	.020	.006*	.062	.051	4.54	1.78	2.23	2.56		2.19	14

I	FA	PCM-R	PCM-1PL	GPCM	GRM	GRM						
		p-value S-X^2				*a*	*b1*	*b2*	*b3*	*b4*	Mean threshold	Rank*
						Digit Span						
F4	.33	.13	.15	.31	.23	.99	-4.83	-1.65			-3.24	F4
F5	.60	.94	.91	.95	.87	1.39	-1.37	-.07			-.72	F5
F6	.66	.29	.19	.43	.45	1.42	-.25	.95			.35	F6
F7	.71	.21	.18	.20	.08	1.61	.63	1.77			1.20	F7
F8	.70	.11	.03	.20	.18	2.06	1.49	2.18			1.84	F8
B3	.26	.15	.03	.73	.56	.57	-7.33	-1.36			-4.35	B3
B4	.61	.48	.31	.39	.40	1.36	-1.11	.36			-.38	B4
B5	.74	.15	.15	.08	.09	1.96	.10	1.13			.62	B5
B6	.77	.18	.15	.03	.14	2.65	.94	1.77			1.36	B6
B7	.75	.07	.047	.02	.054	3.96	1.35	1.96			1.66	B7
B8	.61	.16	.10	.02	.15	4.28	1.70	2.04			1.87	B8
S4	.44	.60	.50	.67	.52	1.06	-2.44	-.15			-1.30	S4
S5	.54	.09	.054	.18	.22	1.24	-.86	.69			-.09	S5
S6	.61	.91	.86	.88	.75	1.33	.97	2.23			1.60	S6
S7	.65	.68	.51	.63	.67	1.91	1.65	2.66			2.16	S7
S8	.61	.16	.10	.34	.33	2.20	1.91	3.24			2.58	S8
						Letter-Number Sequencing						
4	.59	.945	.775	.128	.401	1.23	-3.75	-3.31			-3.53	4
5	.20	.332	.211	.312	.082	1.27	-2.93	-1.41			-2.17	5
6	.07	.244	.184	.938	.283	1.23	-2.30	-.53			-1.42	6
7	.86	.131	.129	.398	.102	1.27	-1.52	-.11	1.43		-.07	7
8	.74	.571	.372	.605	.097	1.23	.48	1.38	2.78		1.55	8
9	.93	.033	.039	.565	.312	3.25	1.22	2.31	2.67		2.07	9
10	.93	.038	.016	.557	.402	2.77	2.23	3.15			2.69	10

Note. *a* = discrimination parameter; *b1- b4* = location parameter for a specific category; Digit Span: F = Forward. B = Backward. S = Sequencing; FA = item loading in factor analysis; PCM-R = partial credit model-Rasch model; PCM-1PL = one-parameter partial credit model; GPCM = generalized partial credit model; GRM = graded response model.

*p < .01
